# Motibot: the Virtual Coach for healthy coping intervention in diabetes

**DOI:** 10.1192/j.eurpsy.2022.453

**Published:** 2022-09-01

**Authors:** G. Bassi, C. Giuliano, A. Perinelli, S. Forti, S. Gabrielli, E. Mancinelli, S. Salcuni

**Affiliations:** 1 University of Padova, Department Of Developmental And Socialization Psychology, Padova, Italy; 2 Fondazione Bruno Kessler, Digital Health Lab, Trento, Italy

**Keywords:** virtual coach, healthy coping, diabetes mellitus, diabetes distress

## Abstract

**Introduction:**

Virtual coaches (VCs) can support people with Diabetes Mellitus (DM) by motivating them to better manage their health. Few VCs were aimed at providing psychosocial support. In this regard, motivation is a pivotal construct in diabetes self-management as it allows adults with DM to adhere to the clinical recommendations.

**Objectives:**

The present study aimed to develop a VC able to motivate adults with DM to adopt and acquire healthier coping strategies, to decrease symptoms of depression, anxiety, perceived stress, and diabetes-related emotional distress, while also improving their well-being.

**Methods:**

A total of 12 adults with DM (M=27.91 years; SD=9.82) interacted with a VC, called Motibot using Telegram for an overall duration of 12 sessions. Participants completed a battery of instruments at pre-, post-intervention and follow-up.

**Results:**

highlighted a decrease in anxiety, and depression symptoms between pre-, post-intervention and follow-up, as also showed by the results that emerged through the text mining. Motibot was perceived as motivating and encouraging in the adoption of appropriate coping strategies, such as mindfulness practices. Motibot was also perceived as trustworthy, reflective, and stimulating in its dialogical interaction. Indeed, adults felt involved in the interaction with Motibot, thereby showing an overall perception of a better quality of life, in the absence of diabetes distress.

**Conclusions:**

This study sheds light on the importance of VCs in health care for people with DM for psychosocial support. This is the first experimental study on the matter, and thus, further iterations of the intervention are needed using a larger sample size.

**Disclosure:**

No significant relationships.

